# Editorial: Repetitive Structures in Biological Sequences: Algorithms and Applications

**DOI:** 10.3389/fbioe.2016.00066

**Published:** 2016-08-04

**Authors:** Marco Pellegrini, Alberto Magi, Costas S. Iliopoulos

**Affiliations:** ^1^Laboratory for Integrative Systems Medicine (LISM), Istituto di Informatica e Telematica, Consiglio Nazionale delle Ricerche, Pisa, Italy; ^2^Laboratory for Integrative Systems Medicine (LISM), Istituto di Fisiologia Clinica, Consiglio Nazionale delle Ricerche, Pisa, Italy; ^3^Department of Clinical and Experimental Medicine, University of Florence, Florence, Italy; ^4^Department of Informatics, King’s College London, London, UK

**Keywords:** repetitive structures, algorithms, tandem repeats, next generation sequencing, transposable elements

**The Editorial on the Research Topic**

**Repetitive Structures in Biological Sequences: Algorithms and Applications**

Repetitive structures in biological sequences are emerging as an active focus of research and the unifying concept of “repeatome” (the ensemble of knowledge associated with repeating structures in genomic/proteomic data) has been recently proposed in order to highlight several converging trends.

One main trend is the ongoing discovery that genomic repetitions are often linked to biologically significant events and functions. For example, an abnormal number of tandem repeating units both in coding and regulatory parts of the genome have been found to cause a series of diseases, including Huntington disease (MacDonald et al., 1993). There are indications of a link between tandem repeat expansion and certain forms of Amyotrophic Lateral Sclerosis (Renton et al., 2011).

Copy Number Variations and alterations (CNV/CNA), not necessarily in tandem, have been demonstrated to be one of the main sources of genomic variation in humans. These participate to phenotypic variation and adaptation and contribute to causing various diseases, including cancer, cardiovascular diseases, HIV acquisition and progression, autoimmune diseases, and Alzheimer’s and Parkinson’s diseases (Zhang et al., 2009).

Genome-wide identification of CNVs can be performed with array-based comparative genomic hybridization (aCGH), SNP arrays, and next generation sequencing (NGS). Although the experimental nature of these technologies is very different, the genomic profiles that they generate for CNVs identification are mathematically very similar. Several computational methods have been published in the last 10 years for segmenting these genomic profiles; however, much work still needs to be done, in particular for discovering CNV in low frequency subclones of cancer samples.

Intragenic tandem repeats polymorphisms may be involved in mis-regulations leading to protein toxicity through multiple pathways. Tandem repeats and CNV in Next Generation Sequencing (NGS) data are, however, difficult to detect and analyze, and devising effective detection algorithms is still a very open area of research (Treangen and Salzberg, 2012).

Repeating structures abound also in human proteins and they are a possible key to exploring sequence, structure, and function relationships. Inverted repeats are fingerprints of DNA hairpins and have been shown to contribute to chromosomal fragility in the human genome.

A second converging trend has been the emergence of many different models and algorithms for detecting non-obvious repeating patterns in strings with applications to genomic data collected in Hight Throughput assays (e.g., reads from NGS sequencing, or assembled genomes). A challenging aspect still to be explored is the full impact of evolutionary sequence divergence, and evolutionary selection over the origin and functional significance of repeating substructure. High divergence repetitions are harder to detect from the genomic background; however, they may give us more insight into the evolution of functional units in the genome. New modeling and algorithmic schemes are emerging to tackle these issues, focusing on the computational characterization of the individual entities involved in the repeatome. Borrowing methodologies from combinatorial pattern matching, string algorithms, data structures, data mining, machine learning, probability, and statistics, these new approaches overcome the limitations of the current approaches and offer an example of trans-disciplinary research.

In this Research Topic, we have collected four original research articles and six reviews spanning the full scope of the Topic.

NGS data are a common theme of three of the contributions. Tattini et al. give an overview of the challenges and the several approaches in the literature for detecting structural variants in the human genome using whole genome and whole exome sequencing data, pointing at major advantages and drawbacks of each approach. Narzisi and Schatz analyze the impact of small-scale repetitive sequences, in particular near-tandem repeats, on the discovery of DNA structural variations with the micro-assembly approach. Manconi et al. describe a GPU-based efficient pipeline for filtering reads obtained from Next Generation sequencing, in conjunction with read depth CNV detection methods.

Repetitive sequences both within a single genome and across multiple genomes cause several problems in building effective genomic databases that support efficient data mining on genomic data. Gagie and Puglisi survey advances in algorithmic techniques for taking advantage of repetitive sequences in indexing and searching genomic databases.

The study of tandem repeats in DNA sequences has been a very active area of research in the last decade. Anisimova et al. survey both computational and statistical approaches for TR detection and their application to sequence alignment, phylogenetic analysis, and benchmarking. Régnier and Chassignet develop new models for predicting the statistics of repetitions and show that the proposed model fits nicely data from a biological case study. Pellegrini gives an overview on the multi-faceted aspects of research on protein tandem repeats (PTR), including prediction algorithms, databases, early classification efforts, mechanisms of PTR formation and evolution, and synthetic PTR design, embracing both sequence and 3-dimensional structural aspects.

Transposable Elements (TE) are DNA subsequences that can replicate themselves via a series of biochemical mechanisms and are particularly abundant in mammalian genomes. Kannan et al. investigate the correlations between TE and long intergenic non-coding RNA genes (lincRNA), corroborating the hypothesis that TE have substantially contributed to the origin, evolution, and functional diversification of lincRNA genes.

Nigita et al. investigate computational aspects of RNA editing, which is a post-transcriptional alteration of expressed RNA sequences eventually affecting protein and ncRNA structure and function. This phenomenon is mostly associated with repetitive regions of RNA sequences.

Besides sequence and 3-dimensional structures, biological data are increasingly available in graphical form. Micale et al. describe a web-based tool (SPECTRA) to build and analyze PPI networks that capture tumor and tissue-specific interactions via integration of a variety of heterogeneous data repositories, thus allowing the comparative exploration of similarities/differences in tissue-specific processes.

This series of papers provides a glance into the rich emerging area of repeatome research, addressing some of its pressing challenges. We believe that these contributions are valuable resources for repeatome research and will stimulate further research from bioinformatic, statistical, and biological points of view.

## Author Contributions

The authors contributed equally to this work.

## Conflict of Interest Statement

The authors declare that the research was conducted in the absence of any commercial or financial relationships that could be construed as a potential conflict of interest.
